# What school-based interventions work to improve attendance in secondary school students with persistent absence? A systematic review

**DOI:** 10.3389/frcha.2025.1603680

**Published:** 2026-01-12

**Authors:** Alice Middleton, Martha Watson, Joanna K. Anderson

**Affiliations:** 1 Department of Public Health and Primary Care, University of Cambridge, Cambridge, United Kingdom; 2 West Suffolk NHS Foundation Trust, Bury St Edmunds, United Kingdom; 3 Department of Psychiatry, University of Cambridge, Cambridge, United Kingdom

**Keywords:** persistent absenteeism, school attendance, school-based interventions, secondary schools, chronic absenteeism, intervention effectiveness

## Abstract

**Background:**

Absence from school is an increasing concern internationally, with significant consequences for children's mental health, academic achievement, and future opportunities. The multifaceted causes of absenteeism, including factors like emotionally based school avoidance (EBSA), have prompted a variety of interventions aimed at addressing this complexity. These efforts include recent plans from the UK government. This systematic review evaluates the effectiveness of school-based interventions targeting persistently absent secondary school students, to inform evidence-based strategies.

**Methods:**

A systematic search was conducted across six electronic databases in health and education for studies published up to April 2024. Eligible studies assessed school-based interventions aimed at improving attendance among persistently absent secondary school students. The Effective Public Health Practice Project (EPHPP) and Critical Appraisal Skills Programme (CASP) tools were used to assess study quality. A bioecological framework was applied to map interventions to influence levels and evaluate their impact on attendance.

**Results:**

Sixteen studies, mostly from the United States with one from Australia, were included. Study designs varied, including randomised controlled trials, quasi-experimental designs, and cohort studies, with quality ratings from weak to moderate. Interventions demonstrated variable effectiveness, reflecting the challenges of addressing persistent absenteeism. Favourable outcomes were reported for mentoring schemes, family involvement initiatives, school counselling, incentive programmes, school-based healthcare, and a police partnership strategy. However, inconsistencies in significance and impact were observed across studies.

**Conclusion:**

The evidence base for interventions to improve attendance among persistently absent secondary school students remains limited. High-quality research is needed to build robust evidence, incorporating comprehensive attendance metrics alongside academic and health outcomes. Future studies should document and analyse demographic subgroups and include qualitative approaches to address the needs of diverse at-risk groups and guide intervention design.

**Systematic Review Registration:**

https://www.crd.york.ac.uk/PROSPERO/view/CRD42024490992, PROSPERO [CRD42024490992].

## 1 Introduction

Consistent school attendance plays a vital role in the individual development of young people and is associated with long-term life chances (1). Higher attendance has been linked to a number of positive outcomes including attendance in continued education, higher earnings, and economic empowerment (2). Consistent attendance is also associated with improved mental and physical health (3), and development of skills in making positive health-related choices (4).

Conversely, lower levels of school attendance are associated with a number of challenges throughout life, including low academic attainment (5–7), low social functioning (8) and increased risk of unemployment (9). Lower attendance is also linked to mental and physical health difficulties (10), including an elevated risk of self-harm and suicide (11). These associations are complex and multi-directional; for some children, reduced attendance may be a response to unmet needs within the school environment, particularly in the context of neurodivergence, emotional distress, or anxiety-based avoidance (often referred to as Emotionally Based School Avoidance, EBSA), and lack of tailored support.

The implications of these outcomes extend beyond the individual, with costly impacts on society. Unemployment and reliance on benefits strain the economy, while poor health outcomes increase the burden on the healthcare system (12). An association with criminal outcomes has also been identified, increasing pressure in juvenile and adult justice systems (13).

In this context, absenteeism can be seen as a pressing public health issue, with the potential to shape individuals’ overall health, well-being, and functional capabilities. Addressing this issue and its drivers, both within and beyond the school environment, will ensure that children can thrive and develop to become part of a healthier community and resilient society.

There are many reasons why students cannot or do not attend school, such as physical and mental health issues, anxiety-related school avoidance, family responsibilities, logistical challenges, social problems, participation in out-of-school activities, and school-related factors (14). The multifaceted nature of the underlying causes of absences poses a challenge in understanding and addressing issues to help students attend (15).

Attendance data in England shows that school absence is a critical issue, particularly when compared with pre-pandemic levels. The overall absence rate increased from 4.7% in 2018/19, to 7.1% in 2023/24. Some pupils miss school at higher rates than others. When absences exceed 10% of possible sessions, this is classified as persistent (or chronic) absence (16), which is associated with greater risk of negative outcomes. Persistent absence rates have risen sharply, from 10.9% of pupils in 2018/19 to 20.0% in 2023/24, with secondary schools experiencing the highest rates of persistent absence.

The absence rate is not uniform across the student population. Using eligibility for free school meals (FSM) as an indicator of disadvantage (17), pupils eligible for FSM were more than twice as likely to be persistently absent (34.8%) compared to their non-eligible peers (14.1%) (18). A similar disparity is seen among pupils with Special Educational Needs (SEN), where 31.3% were persistently absent, compared to 18.4% of those without SEN (18).

Pupils with persistent absence often cite multiple reasons for missing school, and interventions are less likely to be successful (19). For some students, school-related anxiety and distress play a central role, and these patterns can become entrenched if early support is unavailable. If unaddressed, absences may escalate, and can be associated with a higher risk of poor academic achievement and school dropout (20). In the UK, missing over 50% of sessions is known as severe absence (17). 171,269 pupils (2.3%) were classed as severely absent in 2023/24, the highest level recorded.

Rising rates of absenteeism, wide-ranging underlying causes, and the disproportionate burden on disadvantaged groups add complexity to tackling the issue. Addressing school absence effectively requires system-wide interventions informed by public health principles. Given the strong links between absenteeism and wider determinants of health, coordinated efforts are needed across schools, families, health services, policing, and community organisations (21).

In the UK, the Education Committee of Parliament conducted an inquiry into persistent absences in 2023, highlighting uneven distribution and recent increases in absence rates (22). The Government's response includes a “support first” approach, enhanced attendance reporting, designated champions, and multi-agency collaboration across education, health, social care, and policing (23). While these plans build on previous initiatives and stakeholder input, they are limited by a lack of robust evidence.

A 2021 Education Endowment Foundation (EEF) rapid evidence review concluded that most attendance interventions had limited or inconsistent impact, and that few studies were of high methodological quality (24). The review primarily included randomised controlled trials and quasi-experimental designs, which, while rigorous, represented only a narrow subset of the interventions being delivered in schools. A 2022 meta-analysis (25) similarly focused on controlled intervention studies and confirmed that although more interventions are being trialled, the evidence base remains weak and heterogeneous, with no clear consensus on which approaches work best. The EEF review also included interventions with both direct and indirect effects on attendance, such as mentoring, meal provision, and wellbeing programmes, reflecting the multiple interconnected factors shaping school attendance. However, there remains no agreement on which intervention approaches are most effective in different contexts. Together, these reviews highlight the ongoing uncertainty that this review seeks to address.

A 2013 review (26) found that attendance interventions improved truant students' attendance by an average of 4.7 days. It found that collaborative approaches were no more effective than interventions led by single-agencies, supporting a pragmatic approach based on available resources. However, this review focused on truancy and excluded students who were absent due to anxiety or distress. A 2010 review (27) categorised findings according to the level of intervention; student/family-based, school-based, and community-based. This revealed promise for contingency management at the individual level and organisational partnerships at the community level, but they noted that the classification itself had limitations that could misrepresent effectiveness.

Other reviews reinforce limitations of research in this area. A 2018 review (28) found that skills training, family support, and incentive-based strategies often led to improved attendance, but the review lacked quality appraisal, raising concerns about study bias. A meta-analysis of 22 studies (25) found small positive effects for behavioural and academic interventions but noted that many strategies remain understudied or have only minor impacts. Strict inclusion criteria in these reviews often excluded potentially effective approaches that lack precise quantitative measures.

Challenges in studying absenteeism include high family mobility, school disengagement, and inconsistent reporting (26). Standardised evaluation frameworks have been recommended to address inconsistencies (29). Research has also highlighted age-related differences in absenteeism drivers, with younger students dependent on parental involvement and older students facing challenges linked to past academic struggles (28, 30). Older students who have experienced prolonged absence may disengage further, making interventions less effective (31).

Beyond structured interventions, school culture may play a critical role in attendance. Studies link positive school environments to improved engagement (32), and there is growing emphasis on fostering an “attendance-focused” culture (24, 33). Approaches like Multi-Tiered System of Support (MTSS) (34) and Positive Behavioral Interventions and Supports (PBIS) (35) have shown promise in creating supportive environments that encourage attendance. These approaches are increasingly discussed in the context of EBSA for this reason, with potential to reduce sources of emotional distress. Whilst this may strengthen students' sense of safety and belonging, these approaches are implemented inconsistently, and research on their long-term impact remains limited.

Building on this, a recent review of truancy interventions identified that approaches which strengthen students’ sense of connection to school tend to have more sustained effects on attendance. Interventions grounded in a school bonding or relational approach, where the focus is on improving relationships between students, staff, and the wider school environment, were found to be more effective than those that rely primarily on rewards or sanctions directed at students or parents (36). In some cases, sanction-based approaches were shown to be ineffective or counterproductive, particularly for students who were already disengaged. This highlights the importance of considering relational and contextual influences on attendance, rather than treating absenteeism solely as an individual behaviour.

**Figure 1:**
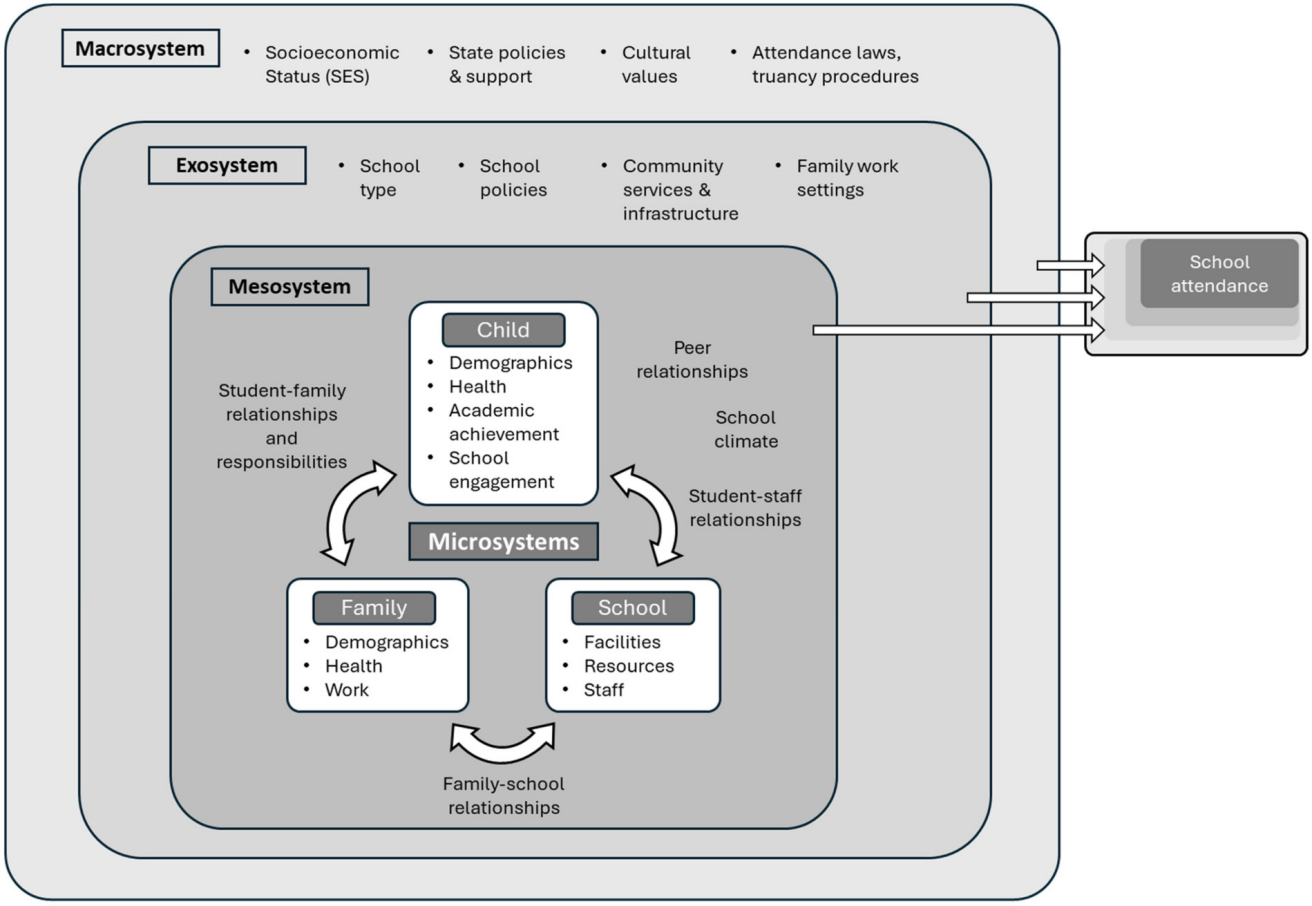
Bioecological framework for school attendance, adapted from KiTES framework (38).

A framework adapted from Bronfenbrenner's theory of human development (37) has been used to identify risk and protective factors for school absenteeism across multiple ecological levels; The “Kids and Teens at School” (KiTeS) framework (38). This conceptualises influences on school attendance at four interconnected levels (as shown in Figure 1):

Microsystem: Direct influences, including individual characteristics, family, and school.
Mesosystem: Interactions between these microsystems, such as family-school relations and school climate.
Exosystem: Indirect influences, including school and community services or parental work settings.
Macrosystem: Broader structural factors, such as socioeconomic status, cultural norms, and public policies.

The framework has been used to structure the identification of both risk and protective factors for absenteeism; for example, one study analysing data from over 121,000 students identified 18 risk and protective variables within the microsystem and mesosystem (40). Another found that interventions often overlooked exosystem and macrosystem factors in elementary school settings, suggesting the need for a more comprehensive approach (41). Additionally, the KiTeS framework has been linked to Multi-Tiered Systems of Support (MTSS) models, incorporating interventions across multiple levels (34). This framework can provide a useful way to consider how different intervention components relate to the wider system influencing attendance.

While various interventions exist, gaps in high-quality, context-specific research persist. The effectiveness of attendance strategies depends on multiple factors, including individual circumstances, school engagement, and broader systemic challenges. Further work is needed to establish which strategies are most effective for different drivers of absence, student populations, and school settings.

Although absenteeism is a global issue, approaches to monitoring, thresholds, and school structures vary internationally. This review focuses on the UK context, but draws on evidence from comparable education systems where international findings may still offer transferable insights.

As understanding of absenteeism evolves, there are increasing calls to shift away from framing non-attendance solely as misbehaviour, instead recognising the broader factors contributing to absence (42). This includes recognising emotionally based school avoidance as a response to distress, and implementing supportive strategies and adjustments.

Given rising rates of persistent absence in secondary schools and the lack of robust evidence on effective interventions, this systematic review evaluates the effectiveness of approaches targeting this group to guide policy and practice. It compares interventions across individual, family, school, community, and societal levels, examining the role of school culture in shaping attendance outcomes. Findings aim to inform UK policies at a time of significant investment in attendance strategies.

## 2 Methods

This systematic review uses the Preferred Reporting Items for Systematic Reviews and Meta-Analyses (PRISMA) guidelines to ensure reporting rigour and transparency (43). The project was registered on an international Prospective Register of Systematic Reviews [PROSPERO] on 09/01/2024, reference ID: CRD42024490992.

### 2.1 Study design and search strategy

The question being addressed is which interventions are most effective in reducing persistent absenteeism and how effectiveness varies across different levels of influence. This is outlined using the PICOS framework: the review focuses on secondary school students (Population), examining interventions designed to address persistent absenteeism (Intervention), with or without a comparator (Comparison), assessing school attendance as an outcome through various measures (Outcome). Only empirical peer-reviewed studies were included (Study Design), ensuring a rigorous evidence base.

The search strategy was developed in Ovid MEDLINE, informed by a review of key publications and refined with input from a librarian to optimise search terms and combinations. Once finalised, it was adapted for use in additional databases. The search incorporated terms related to persistent absenteeism (e.g., “emotionally based school avoidance”, “school refusal”, “non-attendance”), interventions (e.g., “program”, “mentorship”, “counseling”), and school culture (e.g., “school belonging”, “student-teacher relationships”, “classroom environment”). Boolean operators and database-specific subject headings were used to refine the search strategy. The full search strategy is available in the Supplementary Material. Citation and reference list searches were undertaken to explore potential additional studies.

Given the intersection of school absenteeism with both health and education research, multiple databases were searched to ensure comprehensive coverage; PsycINFO (Behavioural Sciences and Mental Health), ERIC (Education Research Information Centre), BEI (British Education Index), Child Development and Adolescent Studies, ASSIA (Applied Social Science Index and Abstracts) and MEDLINE (Medical Literature). The final searches were conducted on 16th April 2024.

### 2.2 Eligibility criteria

Inclusion and exclusion criteria were structured using the PICO framework and informed by pilot searches and background literature. Eligible studies examined interventions targeting persistent absenteeism among secondary school-aged students (aged 11–18), defined using the UK threshold of missing 10% or more school sessions. Studies including mixed-age samples were included where at least 90% of participants met the inclusion criteria or where subgroup data were reported. Interventions designed to proactively support at-risk students were included, and interventions where attendance was a secondary outcome (for example, interventions targeting mental health or behaviour) were included where attendance outcomes were reported.

Studies were required to measure school attendance through any method, including school records, self-reports, or staff reports. Both qualitative and quantitative studies were eligible, provided they included primary data. Although attendance outcomes are often reported quantitatively (e.g., percentage of sessions missed), qualitative evidence was included to provide insight into mechanisms of change, student and staff engagement, and contextual factors influencing acceptability and feasibility. Qualitative data relevant to attendance could include student, parent, or staff descriptions of experiences of returning to school, perceived barriers and facilitators to attendance, and changes in attitudes, confidence, or school connectedness. This supports a more complete understanding of how and why attendance outcomes vary across settings. Studies reporting only feasibility or acceptability without attendance outcomes were excluded.

Commentaries, opinion pieces, and reviews were excluded to avoid secondary interpretation bias, but reference lists of reviews were screened for additional primary studies. Case studies with fewer than five participants were also excluded, as small samples limit the interpretability of findings and restrict generalisability.

The review focused on school-based interventions; therefore, pharmacological treatments, medical interventions, and those delivered in non-school settings were excluded. Given the broad and varied conceptualisation of school culture, the role of culture was assessed in result synthesis rather than used as a strict inclusion criterion.

To ensure relevance to the UK context, studies were limited to countries with comparable compulsory education systems, including Europe, the USA, Canada, Australia, and New Zealand; Comparable systems were pragmatically defined as those with universal compulsory schooling to at least age 16 and broadly similar primary and secondary structures. No date restrictions were applied, but studies were limited to English due to resource constraints.

### 2.3 Study selection

Search results were uploaded and managed using the web-based reference management tool CADIMA (44) for efficient tracking and documentation (45). Duplicate studies were automatically removed using CADIMA and EndNote (46), followed by title and abstract screening to exclude remaining duplicates and obviously irrelevant studies. Screening was conducted by one reviewer, with 10% independently screened by a second reviewer to assess consistency in applying inclusion criteria; agreement exceeded 90%. Any uncertainties at this stage were taken forward to full-text screening.

**Figure 2:**
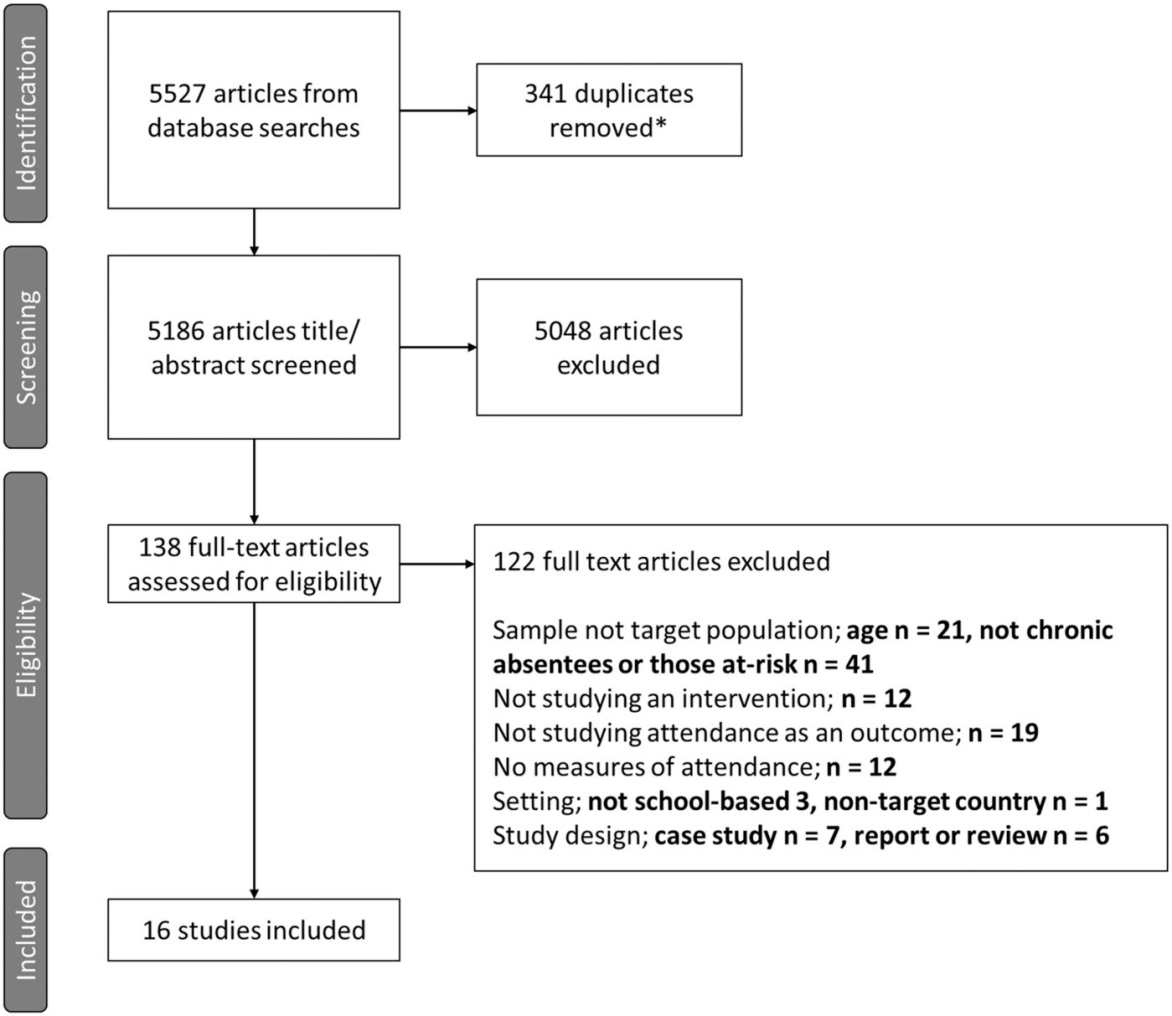
Flowchart illustrating study selection process, according to PRISMA guidelines. *Records marked as duplicates by automation tools, EndNote (46) and CADIMA (44).

Full texts were screened using the same process: one reviewer screened all studies, and a second reviewer independently screened 10% to ensure accuracy and reduce the risk of missing relevant studies (47). Disagreements were resolved through discussion. The screening process is outlined in the PRISMA flowchart (Figure 2).

### 2.4 Data extraction

A spreadsheet was used to extract and organise data from the included studies, with iterative development of categories. The final spreadsheet captured key details, including authors, year of publication, study title, study design, data type, population type, sample size, setting, intervention, comparison, outcome measures, time points of data collection, and findings, which were organised by attendance and other outcomes.

The Template for Intervention Description and Replication (TIDieR) guideline (50) was used to extract further details of interventions. This checklist provides a structured framework for describing interventions, including their rationale, materials and procedures, mode of delivery, personnel involved, setting, timing, and implementation details such as fidelity. These elements facilitated structured comparisons between strategies and their implementation, supporting a comprehensive understanding of the interventions reviewed.

Data extraction was conducted by a single reviewer, with the extraction framework refined iteratively to support consistency across studies.

### 2.5 Critical appraisal

Quantitative studies were assessed using the Effective Public Health Practice Project (EPHPP) Quality Assessment Tool for Quantitative Studies (51), a recommended tool for appraising public health interventions across different study designs (39). This tool evaluates selection bias, study design, confounders, blinding, data collection methods, and participant withdrawals, with each domain rated as strong, moderate, or weak. These ratings determine the overall risk of bias (low, moderate, or high). Two additional components, intervention integrity and analytical methods, further contextualise study limitations.

Qualitative studies were appraised using the Critical Appraisal Skills Programme (CASP) Qualitative Checklist (52), a widely recognised tool for assessing methodological rigour and the relevance of findings in health research (53). This checklist consists of ten prompts across three sections: validity of results, findings, and applicability to practice.

Critical appraisal was conducted by one reviewer, with a sample of studies independently assessed by a second reviewer as a quality assurance measure to ensure consistent application of criteria. A small number of discrepancies were discussed and resolved through consensus, which refined and clarified the application of appraisal criteria before continuing with the remaining studies.

### 2.6 Data synthesis and analysis

Given the diversity in population characteristics, settings, and interventions, a narrative synthesis approach was chosen. This method integrates study results while considering statistical significance, confidence intervals, and risk of bias. A meta-analysis was deemed inappropriate due to the heterogeneity of study designs, which could obscure important contextual factors. Findings were interpreted in relation to study robustness rather than methodological exclusion, acknowledging the complexities of researching a highly mobile population with school engagement challenges (54).

The synthesis followed the Economic and Social Research Council (ESRC) guidelines for narrative synthesis (55) to ensure a systematic and comprehensive analysis. Interventions were grouped through iterative review of study characteristics, identifying common mechanisms of action. Eight categories were developed that reflected the dominant modes of support used to influence attendance [Multifaceted Interventions, Mentoring, Family Involvement, Counselling, School-based Healthcare, Social-Emotional Learning (SEL), Police Partnership and Incentive Schemes].

The KiTeS framework (38) was used to analyse intervention components across the microsystem, mesosystem, exosystem, and macrosystem. Intervention activities were coded to one or more ecological levels depending on their mechanisms of influence. For example, mentoring schemes typically operate at the microsystem through one-to-one relational support, but also influence the mesosystem by facilitating communication and problem-solving between school, family, and the student. These components were therefore coded across both levels. Coded intervention levels were then used to structure the narrative synthesis and to compare the distribution and focus of intervention strategies across studies.

Applying this framework allows an exploration of relationships between interventions and absenteeism, identifying key influences on attendance outcomes. Particular attention was given to school culture within the mesosystem and its interaction with exosystem-level factors such as school policies. The robustness of the synthesis was assessed to account for methodological variability.

## 3 Results

The searches identified 5,527 articles. After title and abstract screening, 138 full-text articles were assessed for eligibility. Of these, 122 were excluded for not meeting the inclusion criteria, leaving 16 studies for the final review, as shown in the PRISMA flowchart, Figure 2.

### 3.1 Study characteristics

The included studies were conducted between 1990 and 2017, with publication dates from 1993 to 2024. Fifteen were based in the US and one was in Australia.

Seven studies were experimental designs (all randomised controlled trials), four were quasi-experimental and five were observational. Thirteen studies collected only quantitative data, while the other three collected both quantitative and qualitative data. One study used a survey to assess students' willingness to attend, the other fifteen studies used school records to measure absence using various metrics. This included days absent, measures of consistent attendance, and percentages of students who were persistently absent. Two studies specifically assessed unexcused absences, and one subdivided absences by specific class attendance.

#### 3.1.1 Participants

Two studies assessed intervention assignment at school-level (schools were classified as implementers or non-implementers). The remaining 14 studies examined individual-level assignment and involved a total of 6,915 participants.

There was notable variability in the reporting of participant demographics, including of characteristics known to be common confounders. Table 1 shows the consistency of reporting for sex, ethnicity, economic disadvantage, and special educational needs (SEN) status across the studies. Only three studies reported all of these demographic characteristics for either the participants or the overall population. SEN status was least frequently reported, with no evidence of measurement or reporting in over half of the studies (9/16), followed by economic disadvantage, which was missing in six of the 16 studies.

**Table 1:** Number of studies which measured and reported demographic characteristics. *N* = 16.

Reporting completeness	Sex (%)	Ethnicity (%)	Economic disadvantage (%)	SEN status (%)
Fully reported	12 (75)	11 (68.75)	4 (25)	4 (25)
Partially reported	0	2 (12.5)	6 (37.5)	2 (12.5)
Measured, but not reported	0	0	0	1 (6.25)
Not reported or measured	4 (25)	3 (18.75)	6 (37.5)	9 (56.25)

Economic disadvantage was assessed by household income or eligibility for subsidised meals. SEN=Special Educational Needs. Fully reported=Studies that reported detailed participant characteristics for control and treatment groups. Partially reported=Studies that reported characteristics for the overall population or school(s). Measured, but not reported=characteristics were included in the analysis or modelling, but not separately reported. Not reported or measured=no evidence that characteristics were recorded or accounted for in the study.

The included studies varied in their design, populations, and interventions. Table 2 provides an overview of the key characteristics, including study design, population, data type, intervention and comparison types, and attendance-related outcomes.

**Table 2:** Characteristics of included studies.

Study	Population	Study design Data type	Number of participants	Intervention type(s)	Comparison type, details	Attendance outcome type and measure
Akos et al. (56)	All students in public schools within a school district	Observational, analytic cohort study Quantitativike	34 schools (2,040 students)	Counselling	Pre-intervention period and non-matched control group; school services as usual	Total absent days in a year Rate of chronic absenteeism (<90% attendance) School records
Barnet et al. (57)	Pregnant students attending an alternative school	Observational, analytic cohort study Quantitative	341	School-based healthcare	Non-matched control group; off-site healthcare	Days absent in a year School records
Converse et al. (58)	Students with a history of, or at-risk of chronic absence at a middle school	Quasi-experimental, pre-post with control group Quantitative	32	Mentoring	Wait-list control group; school services as usual	Number of unexcused absences per student over 18 weeks School records
DeSocio et al. (54)	Students with a history of, or at-risk of chronic absence in a high school with high levels of disadvantage and low academic achievement	Experimental; randomised controlled trial Quantitative	103	Multifaceted approach Mentoring Family involvement School-based healthcare	Control group (and unable-to-enrol group for treatment-on-treated analysis); school services as usual	Days absent, categorised into most-missed and least-missed classes School records
Durham et al. (59)	All students in a school district	Observational, analytic cohort study Mixed	210 schools (211,513 students)	Multifaceted approach Family involvement	Non-matched control group; school services as usual	Percentage of students missing 20 or more days during the school year School records
Gottfredson et al. (60)	All students in a school with high rates of chronic absenteeism, dropout and low academic achievement	Quasi-experimental, pre-post with control group Quantitative	274	Social Emotional Learning	Non-matched wait-list control; school services as usual	Number of days absent during semester School records
Guryan et al. (61)	Students with a history of, or at-risk of chronic absence at a middle school	Experimental; randomised controlled trial Quantitative	1,938	Mentoring Family involvement	Control group; school services as usual	Days absent per year School records
Heppen et al. (62)	Students with a history of, or at-risk of chronic absence across 10 high schools	Experimental; randomised controlled trial Quantitative	553	Mentoring Family involvement	Control group; school services as usual	Percentage of students attending >90% School records
Johnson & Lampley (63)	Students with a history of, or at-risk of chronic absence at a middle school	Quasi-experimental, pre-post without control group Quantitative	57	Mentoring	Pre-intervention period	Mean number of days absent per year School records
Kang-Yi et al. (64)	Students with mental health diagnoses in middle schools within a school district	Observational, analytic cohort study Quantitative	2,617	School-based healthcare	Non-matched control; ex- or never users of school clinic	Percentage of unexcused absent days per year School records
Maynard et al. (65)	Students with a history of, or at-risk of chronic absence at high schools within a school district	Experimental; randomised controlled trial Quantitative	260	Mentoring Family involvement	Control group; school services as usual	Total number of days missed over 9 weeks School records
Mazerolle et al. (66)	Students with a history of chronic absence at schools within a district	Experimental; randomised controlled trial Mixed	102	Police partnership Family involvement	Control group; district services as usual	Student willingness to attend school Survey
McCord et al. (67)	Students with a history of chronic absence in an alternative high school for students who were not able to succeed in traditional education programmes	Observational, analytic cohort study Quantitative	332	School-based healthcare	Non-matched control group; students not registered at school clinic	Percentage absence in school year School records
Powers et al. (68)	Students with a history of, or at-risk of chronic absence in a low-performing high school	Experimental; randomised controlled trial Mixed	54	Mentoring Family involvement	Control group; school services as usual	Days absent from school per year Student records
Sinclair et al. (69)	Students with emotional or behavioural disabilities across 7 high schools in a district	Experimental; randomised controlled trial Quantitative	175	Mentoring Family involvement	Control group; school services as usual	Percentage of students with consistent attendance (no periods of “dropout”, defined as 15 absences within 20 school days) per year School records
Young et al. (70)	Students with a history of chronic absence in a middle school	Quasi-experimental, pre-post with control group Quantitative	77	Incentive scheme Family involvement	Non-matched control group	Monthly attendance percentage School records

The interventions assessed in the included studies were all delivered in school settings, with 11 of 16 incorporating additional home or community-based elements where indicated. Half of the interventions were standalone strategies (56–58, 60, 63, 64, 66, 67), while the other half explicitly linked to external services through referrals or signposting (54, 59, 61, 62, 65, 68–70).

A range of strategies were assessed, involving interventions that can be organised under eight broad categories. Some strategies included elements from multiple categories. The categories were multifaceted interventions, mentoring, family involvement, counselling, school-based healthcare, social-emotional learning (SEL), police partnerships, and incentive schemes. Several interventions incorporated elements from multiple categories. Further details of the intervention characteristics, including their rationale, delivery format, providers, and fidelity assessments, are summarised following the TIDieR framework (42) in Table 3.

**Table 3:** Intervention characteristics [using TIDieR (50) guideline].

Study	Intervention name	Intervention rationale	Material, procedures and delivery	Provider(s)	Duration and frequency	Fidelity assessment
Akos et al. (56)	School counselling, Recognized ASCA Model Program (RAMP) designation	RAMP status: recognition of counselling standards. Counselling: To improve student outcomes in academic, career, and social/ emotional development.	Counselling including individual and group sessions, workshops, and informational materials	School counsellors	1–7 years overall. Detail not reported	Not monitored or recorded
Barnet et al. (57)	Prenatal care in school-based health centre (SBHC)	To provide primary and prenatal care on school site	On-site care including primary, prenatal, delivery, and postpartum care for students; family planning services; primary care for infants and children; parenting education; and mental health services	Family physicians, social worker, part-time psychiatrist, medical assistant, health educator, and receptionist	Up to 3 years of care, at least 9 prenatal visits recommended, but detail not stated	Not monitored or recorded
Converse et al. (58)	Mentoring, school personnel as mentors	Improved student outcomes for at-risk students, including behaviour, attendance and attitude	Regular mentor-student meetings at school. The mentor aimed to build trust and develop effective communication with the student. Mentors modelled honest and ethical behaviours and completed session logs.	Faculty and staff members as mentors	18 weeks; at least one mentoring session per week	Mentor logs from meetings were reviewed to establish what activities were done. The number of meetings ranged from 8 to 22 in the 18-week period; 3 student-mentor pairs every week as planned
DeSocio et al. (54)	Multifaceted: School based health centre (SBHC), mentoring, family involvement	To enhance student engagement for students with a high number of previous absences	SBHC provides comprehensive health services to students on site. Mentors build relationships with students through day-to-day check ins, tutoring, advocacy and support at school. A coordinator also provides check ins and liaises with families, develops a school re-entry plan and helps addresses challenges	Teacher mentors, program coordinator, health centre staff	6 months. Daily student check-ins and one-on-one interactions, two after-school tutoring sessions weekly (duration of activities unclear)	Mentors met with each other and the project coordinator to provide support for implementation. No further details reported
Durham et al. (59)	Full Service Community School strategy (FSCS), “comprehensive support”	To enhance school and community service coordination and effectiveness in supporting students	Highly variable. FSCS coordinator identifies needs and potential partners, engages families, conducts outreach, provides opportunities for involvement, and creates a welcoming school environment	School leadership, FSCS coordinator, school staff, Local community service providers	1–10 years overall. Detail not reported	Not monitored or reported
Gottfredson et al. (60)	Cognitive behavioural instruction class (a “social skills curriculum”)	To develop student skills and reduce problem behaviour, including misconduct and disruptive activities	Lessons from a trained instructor as part of a 27-session social skills curriculum, delivered on site to an established class group during regular school day	Trained instructors	1 semester, a lesson 2 days a week. Intervention repeated for a second group in a second semester.	Instructors kept implementation logs including lesson date, material covered and a perception of how well the students received and completed the lesson. 16 of 27 and 20 of 27 classes were completed, 68% of critical activities were covered in each class. 35% completion of activities overall.
Guryan et al. (61)	“Check & Connect” (“C&C”) mentoring	To reduce truancy, through social capital	One-to-one mentoring in school. Check: Mentors monitor students’ attendance, behaviour, and academic performance. Connect: Mentors establish relationship with students and their families through formal and informal meetings. They intervene to address engagement obstacles. Mentors collaborate with school personnel to achieve outcomes.	C&C mentors and project manager employed by social service agency. Support from research staff	11 months a year for 2 years.	Mentors formally met with students, one-on-one or in small groups, an average of five times a month. More frequent informal check-ins (not detailed). No reports of length/ duration. Home contact through phone calls or visits, twice a month on average but was variable. Variable approaches to mentoring, some acted as tutors from, others acted as case managers, advocates, and counsellors. No further detail described.
Heppen et al. (62)	“Check & Connect” (“C&C”) mentoring	To increase student engagement, performance in school, and school persistence. To promote school completion and prevent dropout	One-to-one mentoring in school. Check: Mentors monitor students’ attendance, behaviour, and academic performance. Connect: Mentors establish relationship with students and their families through formal and informal meetings. They intervene to address engagement obstacles. Mentors collaborate with school personnel to achieve outcomes.	C&C mentors hired by the district A coordinator who was the existing Dropout Prevention Program Manager in the district. Support from research staff	3 years. Weekly student monitoring checks and student meetings at least twice a month.	Monitoring forms were reviewed for data recorded, meeting frequencies, time spent together and student risk status. Check data was recorded 65% of the time in year 1, 46% in year 2 and 33% in year 3. Meetings were held twice weekly in term time for non-transferring students on average. Students who transferred met once a week on average. The scheme was reported to have been implemented with fidelity, except with students who left district schools.
Johnson & Lampley. (63)	Mentoring scheme	To assist at-risk middle school students with school performance or related issues. To establish relationships between at-risk students and caring adults.	Linking Students To Educational Needs; “LISTEN”. Additional support for students outside the regular classroom setting. Emphasis on study habits, interpersonal relationships, problem solving techniques, communication skills, and by encouraging positive behaviours	Mentors included classroom teachers, assistants, counsellors, administrators, custodians, librarians, retired teachers, and cafeteria staff	2 years. Frequency and length of intervention components not reported	Not monitored or reported
Kang-Yi et al. (64)	School-based mental health services	To improve student mental health and academic outcomes	On site mental health services including wraparound services, and outpatient therapy	Health centre staff	2 years. Further detail not reported	Not monitored or reported
Maynard et al. (65)	“Check & Connect” (“C&C”) mentoring	To emphasise education for students, families and teachers, and to reduce and prevent absenteeism, suspensions, failing grades and school withdrawal	One-to-one mentoring in school. Check: Mentors monitor students’ attendance, behaviour, and academic performance. Connect: Mentors establish relationship with students and their families through formal and informal meetings. They intervene to address engagement obstacles. Mentors collaborate with school personnel to achieve outcomes.	Existing coordinators employed as C&C mentors, “monitors”. Support from research staff	6 months. Weekly student meetings, length not reported.	All CIS site coordinators who implemented C&C were trained and monitored to enhance implementation. Not comprehensively monitored or reported
Mazerolle et al. (66)	School engagement programme, police–school partnership	To deter truancy by increasing parental and child awareness of the truancy laws through a collaborative, engaging family group conference (FGC) forum	Coordinated efforts between police officers and the school to engage parents and truanting children. Collaborative meetings were held in schools, community settings or at home. The context of truancy was discussed, including its effects and legal consequences, and an action plan for students was developed and agreed upon	School staff, police officers, trained conference facilitators. Other professionals from mental health services, social workers and family support services	6 months. Average meeting length of 96 min (min = 50, max = 158 min). Follow up contact with families over 6 months, 8.9 contacts were made on average	Staff and the research team monitored treatment fidelity to ensure control participants were not exposed to the intervention. The research team observed meetings, met meeting facilitators and recorded police officer activities on an activity log.
McCord et al. (67)	School-based health centre (SBHC)	To provide adolescent health care and potentially improve academic outcomes such as attendance, suspension rates, withdrawal, and graduation/promotion rates	On site health services provision which included health education, health promotion, screening, and clinical services	Health centre staff, part-time physician, nurse practitioner, clerk, full-time registered nurse, social worker, and clerk.	1 year. No further detail reported	Not monitored or reported
Powers et al. (68)	“Check & Connect” (“C&C”) mentoring	To emphasise education for students, families and teachers, and to reduce and prevent absenteeism, suspensions, failing grades and school withdrawal	One-to-one mentoring in school. Check: Mentors monitor students’ attendance, behaviour, and academic performance. Connect: Mentors establish relationship with students and their families through formal and informal meetings. They intervene to address engagement obstacles. Mentors collaborate with school personnel to achieve outcomes.	Graduate students trained as mentors. Support from research staff.	2.5 years. Once weekly meetings. An average of 20 h meeting time with mentees over 2.5 years (this equates to 13.3 min per week).	Weekly fidelity surveys completed by mentors and biweekly intervention logs provided a basis for feedback from the research team. 19 of 31 students were enrolled for the duration, all treatment participants completed at least one year. Average number of meetings was 46 (range 15–73). The average amount of cumulative “face time” was 20.22 h (range 4–49.5 h). Mentors implemented the intensive intervention 80% of the time when indicated (when a student's risk status changed).
Sinclair et al. (69)	“Check & Connect” (“C&C”) mentoring	To emphasise education for students, families and teachers, and to reduce and prevent absenteeism, suspensions, failing grades and school withdrawal	One-to-one mentoring in school. Check: Mentors monitor students’ attendance, behaviour, and academic performance. Connect: Mentors establish relationship with students and their families through formal and informal meetings. They intervene to address engagement obstacles. Mentors collaborate with school personnel to achieve outcomes.	Specifically hired C&C mentors, “monitors”, and a project coordinator	4–5 years. Weekly contact with mentees. Contact time outside of the school year was about half of what it was during the academic term.	Procedures were developed to sustain treatment fidelity including coordinator reviews of monitoring sheets. Intervention contacts were recorded to assess engagement. Meetings ranged from a couple of minutes to several hours. On average this was a little less than an hour total a week. Details not reported.
Young et al. (70)	Attendance reward scheme “Perfect Attendance Wins Stuff” (“PAWS”)	To improve the attendance of students who are chronically absent	Daily student check-ins, group activities to promote school bonding, weekly and monthly celebrations and prizes for students. An additional individualised approach for those not improving, to include outreach and education to parents, and referrals to community agencies to address barriers to school attendance.	School staff including social worker, counsellor, psychologist, administrators, nurse	At least 1 month, up to 3 months. Daily check-ins, weekly and monthly perfect attendance celebrations. Duration not reported.	25% of students had additional individualised support. No fidelity monitoring, no further detail reported

#### 3.1.2 Reported outcomes

Across the included studies, outcomes primarily measured student attendance, academic performance (grades, class failure), school engagement, and discipline (suspensions, referrals). Several studies also included qualitative or indirect indicators related to student experience, such as school attitude, reflections on mentoring, willingness to attend, and self-reported adjustment. These outcomes were reported inconsistently and were not systematically extracted, so they are not synthesised in detail. Nevertheless, they may reflect aspects of emotional wellbeing, confidence, or engagement that underpin attendance improvements and highlight areas for future research.

### 3.2 Quality assessment

All studies were assessed using the EPHPP quality appraisal tool (51), with no studies achieving a global rating of “strong”. Eight were rated “moderate” and eight were rated “weak”.

**Figure 3:**
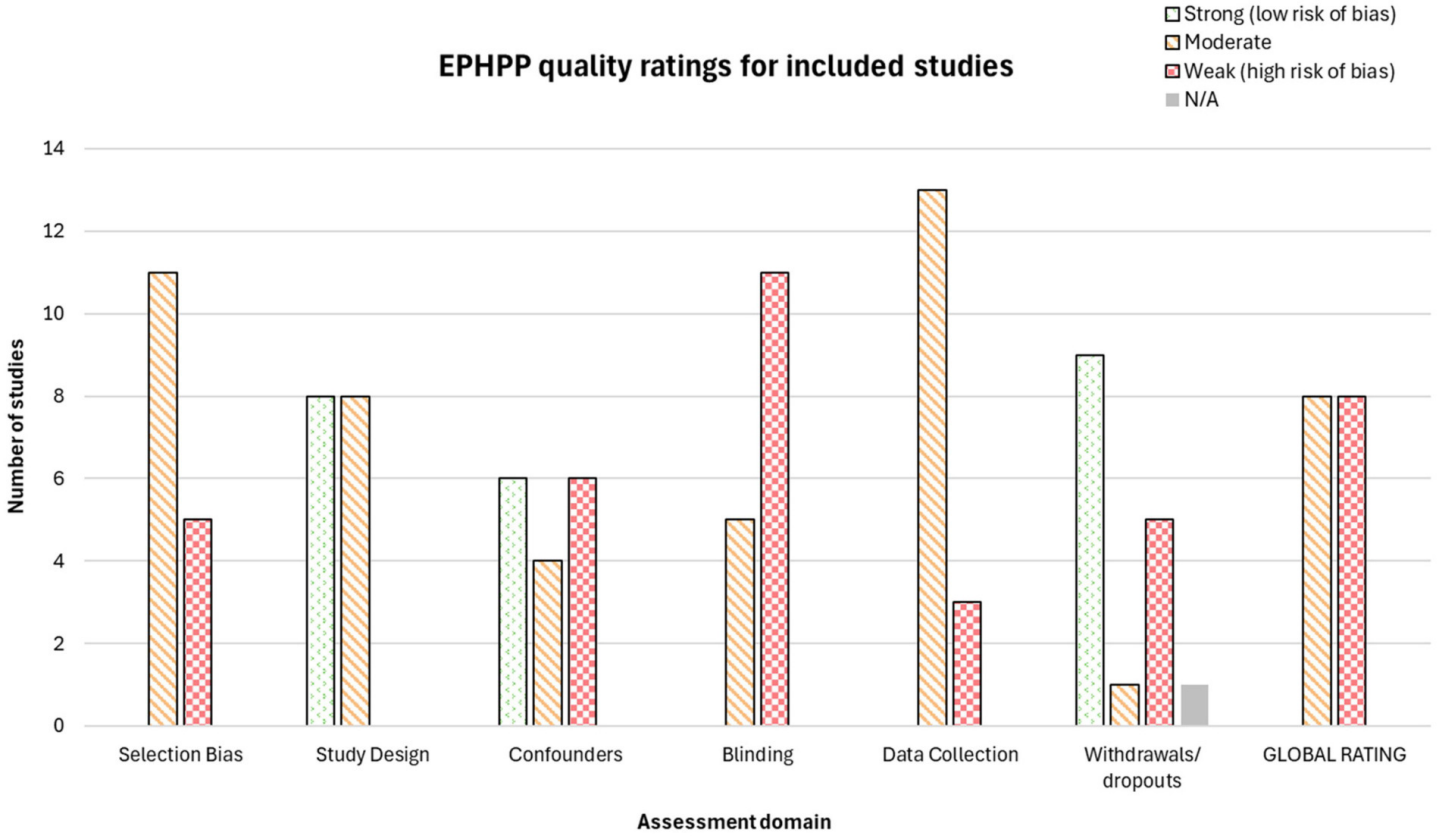
EPHPP quality ratings across assessment domains for included studies.

This is summarised in Figure 3. Assessments of each study are detailed in Tables 4, 5.

**Table 4:** EPHPP quality ratings.

Study	Selection bias	Study design	Confounders	Blinding	Data collection	Withdrawals/dropouts	Global rating
Akos et al. (56)	M	M	W	M	M	N/A	Moderate
Barnet et al. (57)	M	M	M	M	M	W	Moderate
Converse et al. (58)	M	M	M	W	M	S	Moderate
DeSocio et al. (54)	W	S	S	W	M	S	Weak
Durham et al. (59)	M	M	M	M	M	W	Moderate
Gottfredson et al. (60)	M	S	M	W	M	M	Moderate
Guryan et al. (61)	M	S	S	W	M	S	Moderate
Heppen et al. (62)	W	S	S	W	M	W	Weak
Johnson & Lampley (63)	M	M	W	W	M	S	Weak
Kang-Yi et al. (64)	M	M	W	M	M	S	Moderate
Maynard et al. (65)	M	S	S	W	M	M	Moderate
Mazerolle et al. (66)	W	S	W	W	W	S	Weak
McCord et al. (67)	M	M	W	M	M	W	Weak
Powers et al. (68)	W	S	S	W	M	S	Weak
Sinclair et al. (69)	M	S	S	W	W	S	Weak
Young et al. (70)	W	M	W	W	M	W	Weak

S, strong; M, moderate; W, weak.

**Table 5:** CASP quality ratings.

Study	Validity						Ethics & findings			Relevance
Durham et al. (59)	Y	Y	Y	Y	N	N	N	N	Y	Y
Mazerolle et al. (66)	Y	Y	N	Y	N	N	N	Y	Y	Y
Powers et al. (68)	Y	Y	Y	Y	Y	N	N	Y	Y	Y

The included studies had significant sources of bias, with common weaknesses in selection bias, blinding, and intervention integrity. No studies were rated as strong for selection bias, as participants were often not fully representative of the target population due to stratified sampling, risk-based selection, or exclusion of key groups. Recruitment challenges were also prevalent, with some studies reporting extended start-up periods and low participation rates.

Study design was rated strong for eight controlled trials and moderate for quasi-experimental and observational studies. Confounding risk varied; studies rated strong demonstrated no baseline differences between groups in key demographics such as sex, ethnicity, income, and education status. In contrast, weaker studies lacked baseline data or reported significant group differences.

Blinding was the most frequently rated weak domain, primarily due to unclear participant awareness of study aims and insufficient reporting on assessor blinding. None of the experimental studies detailed whether participants were informed of research aims, increasing the risk of reporting bias. Additionally, outcome assessors, often school staff, may have been aware of intervention status, leading to potential detection bias.

Data collection was also a concern, particularly for attendance measures, which were drawn from school records in most studies. While considered valid, such records may not always accurately reflect student presence due to tardiness, selective attendance, or procedural inconsistencies. No studies provided reliability checks for attendance data, leading to cautious assessments of validity.

Handling of withdrawals and dropouts varied, with nine studies rated strong for reporting attrition by group and maintaining over 80% completion rates. Some studies did not report dropouts, while others had attrition rates exceeding 20%, raising concerns about data completeness and potential bias.

Intervention integrity was inconsistently reported, with 12 studies failing to measure consistency, and nine acknowledging possible unintended intervention. Statistical analysis methods were generally appropriate, with nine studies following an intention-to-treat approach.

Overall, all studies presented some risk of bias, often linked to challenges faced by at-risk populations, such as conflicting priorities, low school engagement, and circumstantial barriers. Insufficient reporting undermined the certainty of quality assessments. These limitations are consistent with challenges reported in other school-based intervention research and are considered in the interpretation of results.

The qualitative findings from the three mixed studies were appraised using the CASP tool, which revealed potential limitations in validity, and consistently identified a lack of reporting on ethical issues, as shown in Table 5. The aims of this review were not necessarily addressed by all qualitative aspects of these studies, so further examination of limitations is detailed according to relevance in the synthesis.

### 3.3 Main findings

The level influence of most interventions was individual, focusing on the needs and characteristics of students. For instance, mentoring and counselling schemes provided direct student support, incentives aimed to increase individual student motivation, and healthcare services supported individual health. Some interventions also targeted the family level of influence, through improving communication and links between schools and families, although no interventions explicitly targeted family dynamics or culture as a primary aim. There were no interventions that explicitly addressed school level in the interventions, with no direct strategies to influence the school environment. At the community level, there was involvement of community services within several interventions, and a police-school partnership represented an example of collaborative efforts (66). These initiatives still primarily served individuals, whilst recognising the importance of community support in addressing school attendance issues. Finally, at the societal level, one multifaceted approach advocated for creating a supportive societal environment for schools (59). Links to community services represented a potential role for society as a whole, but again, these were mainly focused on supporting individual students.

Given this distribution with a heavy emphasis on individual-level approaches, results are presented according to the eight intervention categories previously outlined. Table 6 provides a summary of outcomes, grouped by these categories. Aspects of the interventions will be applied to the bioecological KiTES framework (38) to enable an understanding of their outcomes, which is further explored in the discussion.

**Table 6:** Summary table; study, intervention and outcome synthesis.

Reference	Intervention name	Intervention type(s)	Outcome	Test used Significant effect?*	EPHPP rating
Multifaceted approaches					
DeSocio et al. (54)	Multifaceted: School based health centre (SBHC), mentoring, family involvement	Multifaceted approach Mentoring Family involvement School-based healthcare	Days absent categorised into most- and least- missed classes over 9 weeks Most-missed classes: Control: 34.4 days, Intention to treat (ITT): 31.3 days (−3.1 days) *p* = 0.23, Treatment on the treated (TOT): 28 days (−6.4 days) *p* = 0.047. Least-missed classes: Control: 9.4 days, ITT: 7 days (−2.4 days) *p* = 0.08, TOT: 4.5 days (−4.9 days) *p* = 0.004.	ANOVA **Yes, reduced absence**	Weak
Durham et al. (59)	Full Service Community School strategy (FSCS), “comprehensive support”	Multifaceted approach Family involvement	Percentage of students chronically absent (missing >20 days in a school year) - no clear patterns of difference between high schools with and without the FSCS strategy over time of study period.	Visual representation and trend observations **No**	Moderate
Mentoring					
Converse et al. (58)	Mentoring, school personnel mentors	Mentoring	Mentored students had 6.69 unexcused absences over 18 weeks on average, whilst control students had 9.06 (mentoring, −2.37 absences). *p* = 0.177	ANOVA **No**	Moderate
Guryan et al. (61)	“Check & Connect” (“C&C”) mentoring	Mentoring Family involvement	Cohort 1: (*n* = 1,938). Compared with control: ITT 2.29 fewer days absent per year, TOT 4.22 fewer days absent, *p* < 0.01. Cohort 2: (*n* = 1,078). Compared with control: ITT 1.92 fewer days absent per year, TOT 4 fewer days absent, *p* < 0.05.	Regression analysis **Yes, reduced absence**	Moderate
Heppen et al. (62)	“Check & Connect” (“C&C”) mentoring	Mentoring Family involvement	Percentage of students attending >90% in a year Control 73.1% (*n* = 146), Treatment 81.6% (*n* = 146) - Estimated difference 8.6% (regression analysis), *p* = 0.120.	Regression analysis **No**	Weak
Johnson & Lampley (63)	Mentoring, “LISTEN” (Linking Individual Students To Educational Needs)	Mentoring	Mean number of days absent per year: Pre-post: 37.4–27.2 days absent = 10.28 fewer days (*n* = 54), *p* < 0.001.	Paired *t*-test **Yes, reduced absence**	Weak
Maynard et al. (65)	“Check & Connect” (“C&C”) mentoring	Mentoring Family involvement	Total number of days missed over 9 weeks, treatment: 0.577 fewer days absent (*n* = 189), *p* > 0.1.	Hierarchical linear modelling **No**	Moderate
Powers et al. (68)	“Check & Connect” (“C&C”) mentoring	Mentoring Family involvement	Days absent from school per year; *p* = 0.037 Control (*n* = 20): Sixth grade absences (pre) = 14.7, eighth grade absences (post) = 21.6 (+6.9) Treatment (*n* = 25): Sixth grade absences (pre) = 11.08, eighth grade absences (post) = 11.04 (−0.04)	ANCOVA **Yes, reduced absence**	Weak
Sinclair et al. (69)	“Check & Connect” (“C&C”) mentoring	Mentoring Family involvement	Percentage of students with consistent attendance per year (no periods of “dropout”, defined as 15 absences within 20 school days) Year 3 (*n* = 144): Control 30%, treatment 39% (+9%), *p* = 0.037 Year 4 (*n* = 144): Control 30%, treatment 41% (+11%), *p* = 0.001 Year 5 (*n* = 29): Control 6%, treatment 33% (+27%), *p* = 0.031	Chi-squared **Yes, reduced absence**	Weak
Counselling					
Akos et al. (56)	School counselling, Recognized ASCA Model Program (RAMP)	Counselling	After RAMP designation: All students: 0.325 fewer days absent in a year, *p* < 0.05. Economically disadvantaged students 1.7 percentage points less likely to be chronically absent, *p* < 0.05. Hispanic students 1.9 percentage points less likely to be chronically absent, *p* < 0.05.	Regression modelling **Yes, reduced absence in subgroup analysis**	Moderate
School-based healthcare					
Barnet et al. (57)	Prenatal care in school-based health centre (SBHC)	School-based healthcare	Students using SBHC had 12 fewer days absent during year of pregnancy compared with those not (60 vs. 72 days). *p* < 0.01	Independent samples *t*-test **Yes, reduced absence**	Moderate
Kang-Yi et al. (64)	School-based mental health services	School-based healthcare	Percentage of unexcused absent days per year, change over 3 years: ongoing clinic use +4.5% (3.6%–8.1%), stopped clinic use + 7% (9.2%–16.2%), never used clinic +5.2% (11.2% to 16.4%), *p* < 0.001.	General linear regression **Yes, reduced absence**	Moderate
McCord et al. (67)	School-based health centre (SBHC)	School-based healthcare	Percentage absence in school year; those who used SBHC- 35%, those registered who did not use SBHC- 51%, those not registered- 41%, *p* < 0.02.	Chi-squared test **Yes, reduced absence**	Weak
Social emotional learning					
Gottfredson et al. (60)	Cognitive behavioural instruction class (social skills curriculum)	Social Emotional Learning	Fall semester groups, log adjusted days absent on average: during semester, treatment 1.1, control 1.0 – non-significant. At the end of the year, treatment 0.9, control, 0.8 – significant. Spring semester groups, during semester, treatment 0.9, control 0.8 – non-significant.	ANCOVA **No**	Moderate
Police partnership					
Mazerolle et al. (66)	School engagement programme, police–school partnership	Police partnership Family involvement	Student willingness to attend from survey data. The intervention significantly increased willingness to attend (*b* = 0.645, *p* = 0.002).	Multiple regression **Yes, reduced absence**	Weak
Incentive scheme					
Young et al. (70)	Attendance reward scheme “Perfect Attendance Wins Stuff” (“PAWS”)	Incentive scheme Family involvement	Monthly attendance percentage Comparison; no change in attendance across 4 months PAWS; Attendance increased from baseline of 82.4% to 94.6% in first month, (+12.2%) *p* < 0.001. Attendance in the second and third months was 92% and 91.9% respectively.	ANOVA **Yes, reduced absence**	Weak

* A significant effect is based on an alpha level of *p* < 0.05, regardless of the threshold chosen by individual studies.

#### 3.3.1 Multifaceted approaches

Two studies examined multifaceted interventions, integrating academic support, mental health services, family engagement, and community partnerships to provide holistic student support, with mixed results on absenteeism.

The first study (59) evaluated Full-Service Community Schools (FSCS), an integrative strategy combining educational, health, social, and community services within schools and the broader community (71). FSCS aligns with multiple system levels of the KiTES framework (38). At the microsystem level, it provided direct support to students and families, including counselling, health services, and academic assistance. The mesosystem level focused on strengthening school-family collaboration through parent engagement programmes and communication initiatives. Community integration was a key component at the exosystem level, offering access to local health clinics, social services, and after-school programmes. Finally, at the macrosystem level, FSCS promoted policies aimed at achieving educational equity.

The study compared absenteeism in six FSCS schools and 49 non-FSCS schools, noting that FSCS schools had higher pre-existing levels of persistent absence due to greater economic and educational needs. However, no consistent attendance improvements were observed post-implementation. The study did not conduct statistical tests, limiting its ability to assess the intervention's impact. A mixed linear regression analysis combined primary and secondary school data, making it less applicable to this review's focus. Qualitative findings from high-performing schools highlighted the importance of fostering positive family-school relationships and a welcoming school environment, with existing school staff playing a key role in student support.

The second study (54) tested an intervention combining mentoring, school-based healthcare, and family involvement using a randomised controlled trial (RCT). This can again be applied to system levels of the KiTES framework. At the microsystem level, mentoring helped students engage with their education by fostering personal interests, while coordinators provided direct support to both students and families, including re-entry planning. Access to school-based health centres (SBHCs) ensured students received personal and medical support. At the mesosystem level, collaboration between mentors, coordinators, and school staff created a network of support to address barriers to engagement. The exosystem level involved connecting families to community resources, including transport assistance, to improve accessibility. While the intervention did not implement direct macrosystem-level strategies, it contributed indirectly to broader systemic changes supporting student success.

The intervention aimed to strengthen student-school connections. Recruitment challenges led to analysis across three groups: intervention, “unable to enrol”, and control. Both intention-to-treat and treatment-on-treated analyses assessed its effectiveness. Attendance was analysed by class to capture selective attendance patterns.

Over nine weeks, intention-to-treat analysis showed a near-significant improvement in attendance for classes students least often missed (6.96 vs. 9.4 days absent, *p* = 0.08). Treatment-on-treated analysis revealed significant differences; for classes that students least often missed, the intervention group missed 4.5 days over nine weeks, compared to 9.4 days in the control group (4.5 days fewer, *p* = 0.004). For classes that students most often missed, the intervention group missed 28 days, compared to 34.4 days in the control group (6.4 days fewer, *p* = 0.047). Although percentages were not calculated, these figures suggest attendance improvements of approximately 10.9% and 14.2%; this assumes nine five-day weeks, however, which was not clearly specified, and therefore may not be accurate.

#### 3.3.2 Mentoring

Mentoring aimed to prevent dropout, improve academic performance, reduce truancy, and support at-risk students through personalised relationships. Some studies also sought to enhance student engagement, with one explicitly aiming to build social capital (61). Of the eight mentoring studies, five evaluated “Check & Connect” (C&C) programs and three evaluated non-C&C programmes. C&C mentoring programs generally had a primary focus on attendance, whereas non-C&C programmes aimed broadly at supporting at-risk students' engagement, behaviour, and attitudes, as well as attendance. Of the eight studies on mentoring, five found a significant positive impact on attendance, while three showed no effect; significant outcomes were not aligned with whether programmes were C&C or non-C&C.

Longer interventions with high participant retention appeared to be more effective; of the studies without significant findings, two had shorter durations of 18 weeks (58) and six months (65), and the third lasted three years (62) but had the highest attrition rate (47.2%). Among the five studies with significant effects, reductions in absenteeism ranged from 2 to 10 days per year, equating to a 1.1%–5.7% attendance increase over a 175-day US school year. The qualitative findings in a mixed-methods study on “C&C” (68) highlighted implementation challenges, including student mobility, missed sessions, and institutional barriers. This provided insight into variable programme delivery. Persistent and proactive mentorship helped mitigate these issues.

As expected given their broader aims, non-C&C programmes show more variable effects on absenteeism, with reductions primarily observed when interventions are intensive or long-term. However, among C&C programs, outcomes were not uniform, reflecting the potential influence of factors such as implementation fidelity, intensity, and contextual conditions.

#### 3.3.3 Family involvement

Family involvement was a component of five mentoring-based interventions (61, 62, 65, 68, 69), typically through mentor communication via phone calls or home visits. Two multifaceted interventions also incorporated family engagement but used broader approaches. For instance, one involved families in “student re-entry plans”, which included restructuring routines and providing practical resources like transport tokens and alarm clocks (54). Another (59) aimed to understand attendance barriers within family life, addressing issues such as nutrition and unstable housing while fostering school-family engagement through volunteering opportunities. Across these studies, family involvement ranged from largely one-way communication from school to home, to more collaborative approaches in which families actively participated in problem-solving. However, because family involvement was rarely evaluated independently, it was not possible to determine whether communication-focused or partnership-based models were more effective. Outcomes should therefore be interpreted in the context of the wider intervention components rather than family involvement alone.

One study (66) placed family involvement at the core of its truancy strategy, using “family group conferences” to align students, families, schools, and police on truancy laws. A survey assessing student willingness to attend school found a significant treatment effect (*b* = 0.645, *p* = 0.002). The intervention was most effective when parents better understood their legal responsibilities.

Another study (70) integrated family engagement into an incentive programme, using calls, letters, and home visits for targeted support and referrals to community services. Families reported feeling supported by the school, and student attendance increased significantly from 82.4% to 91.9–94.6% over three months (*p* < 0.001), while the control group showed no change. This improvement occurred alongside other intervention components, which are discussed below.

#### 3.3.4 Counselling

One study assessed the effect of counselling by analysing student outcomes based on schools' accreditation with the Recognized ASCA Model Program (RAMP) from the American School Counselor Association (ASCA) (56). RAMP accreditation signifies the implementation of comprehensive counselling services aimed at improving academic, career, and social/emotional development (72). The RAMP program also advocates for a comprehensive approach to support all students, enhancing school culture and contributing to school improvement plans. The study examined the impact of RAMP status on student attendance and achievement.

Results showed that middle school students were absent about one-third (0.325) of a day less per year after RAMP accreditation (*p* < 0.05). The study also assessed the impact on persistent absenteeism, while no significant difference was found across the entire student population, subgroup analysis showed that Hispanic students were 1.9% less likely to be persistently absent, and economically disadvantaged students (eligible for subsidised meals) were 1.7% less likely to be persistently absent post-accreditation (*p* < 0.05 for both).

#### 3.3.5 School-based healthcare

Of the four studies examining provision of health services for students, two were general health clinics. Registration with a school-based health centre (SBHC) was part of a multifaceted intervention (54). An observed reduction in absenteeism (4.9–6.4 fewer missed days a year) cannot be definitively attributed to SBHC involvement without more comprehensive data. The study highlighted a high prevalence of health needs among absent students, and SBHC staff provision was increased to provide support, but data on registration and usage was not captured.

The second study, at an alternative US high school (67), compared students who used the clinic with those who didn't. No overall link was found between clinic registration and absenteeism, but students who used the clinic had 36% absenteeism, compared to 51% for those who registered but didn't use it, and 41% for non-registrants (*p* < 0.02).

Two other studies focused on specific health services. Pregnant students using school-based prenatal care had 12 fewer days of absence than those using non-school-based care (60 vs. 72 days, *p* = 0.001) (57). In another study, students with a mental health diagnosis who continued to use school mental health services had the smallest increase in absenteeism over three years, with significant differences compared to those who stopped using, or never used the services (*p* < 0.001) (64).

#### 3.3.6 Social emotional learning

One study explored the impact of group cognitive behavioural instruction classes aimed at improving social skills and reducing problem behaviour (60). Classes were held twice weekly for a semester, with a second group receiving them in the following semester. Implementation faced challenges, including frequent absences among students and staff. The first semester saw 68% of critical activities delivered, while only 35% were delivered in the second semester.

In the first semester, there were statistically significant improvements in positive peer associations, victimisation, and rebellious behaviour. However, for attendance, students in the intervention group had more days absent compared to the comparison group by the end of the year (log-transformed data: 0.9 vs. 0.8 days absent respectively), though the analysis was limited due to incomplete data and student dropouts. No significant differences were found between the groups in the second semester and at other measurement points.

#### 3.3.7 Police partnership

A structured police-school partnership intervention aimed at improving understanding of truancy laws in Australia, with family involvement through parental engagement and co-produced solutions with students (66). Post-intervention, students showed increased willingness to attend school, with this effect moderated by increased parental awareness of truancy laws achieved through collaboration.

#### 3.3.8 Incentive scheme

The “Perfect Attendance Wins Stuff program”, implemented in a US middle school, targeted students with low attendance through group activities, regular check-ins, and recognition with prizes and celebration meals (70). Ten of the 41 students received additional support, including family involvement and referrals. The programme resulted in a significant increase in attendance, from 82.4% at baseline to 94.6% during participation, which was sustained at 92% for the two months following the intervention, when participation was optional. A breakdown of participation duration was not reported.

#### 3.3.9 Other intervention features

Across all 16 studies, there was no consistent pattern in how additional intervention features influenced effectiveness. The success of mentoring programmes with longer durations was not mirrored in other categories. For instance, a one-month incentive programme showed a significant effect on attendance (70), while a multifaceted intervention over 2–10 years did not (59). There was no clear link between fidelity monitoring and effectiveness; nine studies without fidelity monitoring showed positive attendance effects, while three that did monitor fidelity found no significant effects. Reporting was not consistent enough to determine if fidelity itself was an influential element for effectiveness.

Similarly, no clear correlation was found between the type or number of staff involved, their roles, or the provision of additional training. Four studies with research staff support had mixed results on attendance. Group-based interventions showed varied results, with one study showing a significant positive effect, while the other did not. The remaining 14 individual interventions also had mixed outcomes. In summary, none of these features consistently influenced intervention success.

Notably, none of the included studies explicitly assessed adverse or unintended effects. While several studies reported behavioural or social adjustment measures, these were framed as indicators of positive change rather than potential harms. As a result, it is unclear whether increased attendance may have had negative consequences for some students, for example where school environments felt unsupportive, or exacerbated stress or anxiety.

## 4 Discussion

The aim of this study was to systematically review the effectiveness of interventions that address persistent school absenteeism in secondary schools. Sixteen studies were included, which used diverse strategies to address student attendance. Weak to moderate evidence was identified, with no studies providing strong evidence. Levels of influence, from the individual to the broader society, were only covered to a limited extent. No evidence directly addressed the effectiveness of interventions to reduce persistent absenteeism through targeting school culture. While some interventions showed effectiveness, few consistent findings emerged across intervention categories or common features.

### 4.1 Key findings and existing literature

The effectiveness of multifaceted intervention strategies showed mixed results. These interventions, which align with the bioecological model to target multiple system levels, are gaining attention due to their potential to address the complex and diverse needs of students (34). However, the evidence supporting their effectiveness remains limited and inconsistent, particularly because of short durations, single-site implementation, and lack of detailed reporting of intervention components. While some studies support the use of these approaches (34, 73, 74), others have failed to demonstrate their superiority over simpler interventions (26).

A significant limitation in interpreting findings here is the lack of detail regarding the individual components of the intervention strategies, coupled with insufficient fidelity monitoring and limited efforts to mitigate or document contamination. This issue was particularly evident in the investigation of Full-Service Community Schools (FSCS), where the bespoke nature of each school's approach, based on specific needs, made it difficult to draw definitive conclusions. Although qualitative data from a small sample of schools provided valuable insights into what might make certain strategies effective, the credibility of these findings was compromised by concerns during quality appraisal. The researcher-participant relationship was not explored, and the philosophical stance and positionality of the researchers were not addressed. The analysis was described only as a “qualitative analysis using NVivo software”, with no further detail around the approach used (59).

It is unclear which strategies were used by schools with less successful outcomes, making it difficult to confidently attribute positive outcomes solely to the strategies discussed. Without stronger evidence of variation in strategy implementation and its corresponding effects, assessing how FSCS influenced students across the six implementing high schools becomes challenging. This also limits our ability to identify which system levels of the bioecological model were engaged by the intervention. Additionally, non-FSCS schools may have adopted similar strategies, further complicating the understanding of outcomes and the FSCS approach.

A second multifaceted strategy showed some promise in improving attendance, and provided valuable evidence of selective attendance, revealing a wide variation in how a student may attend between different classes (54). It also offered insights into the potential impact of implementation factors, with significant differences detected in the per-protocol analysis but not in the intention-to-treat analysis. However, the study was single-site with a short duration (nine weeks), limiting depth and confidence of conclusions.

Overall, the two multifaceted interventions included in this review showed mixed effects on attendance, highlighting that evidence for this approach remains inconclusive.

This review found weak to moderate evidence supporting mentoring as an effective strategy to improve attendance for at-risk students, with five of the eight mentoring studies demonstrating significant reductions in absenteeism, particularly when programmes were sustained over longer periods. Mentoring provides individualised support to students with poor attendance, fostering personal relationships and guidance in areas like communication and study skills. These interventions primarily operate within the microsystem of the bioecological model, with direct student-mentor interactions. Mentors also influence the mesosystem by connecting students, families, and school personnel. For example, mentors can advocate for students, helping resolve problematic student-teacher relationships, while also working with families to emphasise the importance of education and prioritising school. Such mesosystem interactions contribute to a more supportive learning environment that may help re-engage absent students, including those experiencing emotionally based school avoidance, where relational trust and reduced anxiety are key to re-engagement.

One study highlighted the importance of mentor-mentee rapport as a key implementation factor in the effectiveness of mentoring (68), with institutional barriers also playing a role. However, a previous meta-analysis of school-based mentoring did not establish a significant effect on attendance (75). The discrepancy may lie in the specific populations targeted in this review, where mentoring was focused on at-risk students, rather than a broad approach. Mentoring may be particularly effective in addressing barriers to attendance, academic struggles, or personal issues. Building trust and rapport with a mentor could provide a positive school relationship for at-risk students, potentially boosting engagement and attendance (76).

While mentoring theoretically could influence a student's exosystem, through mechanisms such as work with policymakers or school administrators, this was rarely discussed in the studies. Similarly, the potential impact on cultural norms or societal values in the macrosystem was not a primary goal of the mentoring programmes described.

In conclusion, mentoring primarily operates at the microsystem and mesosystem levels of the bioecological model and may be an effective intervention for at-risk students when sustained over a long duration. However, the evidence is inconclusive. Barriers such as lack of universal school staff support and institutional policies may hinder its effectiveness. Successful mentoring programmes can foster supportive relationships, increase student engagement, and potentially improve school culture, but there is no conclusive evidence to confirm these effects.

Family involvement was a key feature in five of the mentoring interventions and was also present in other strategies, which showed weak to moderate evidence of effectiveness in reducing absenteeism. Previous research has highlighted the value of family engagement in addressing absenteeism (77), and the evidence in this review supports this. Interventions that facilitated direct contact between schools and families have been found to be particularly effective (78).

Family involvement operates at the microsystem and mesosystem levels of the bioecological model. While mentoring programmes sought to increase communication with families, other studies placed greater emphasis on community links and practical support, contributing to a more comprehensive approach. This model, described as “overlapping spheres of influence”, sees the family, school, and community working together to support a student (79). Collaborative efforts from community partners create a support network that can enhance the effectiveness of interventions. A similar approach is seen in Full-Service Community Schools (FSCS), which have shown promise in building community partnerships and reducing persistent absenteeism in elementary school students (79). However, there was no clear pattern of effectiveness based on the extent of family involvement in the studies reviewed here.

The exosystem was less directly engaged, although sharing information between families and external parties could potentially impact factors like workplace policies or community resources (80). This potential was not explored in the included studies, limiting the interpretation of broader societal effects of family involvement.

This review found evidence suggesting that accreditation with national counselling standards in the US may reduce persistent absenteeism in some student groups. One study reported a 1.9% reduction in absenteeism for Hispanic students and a 1.7% reduction for economically disadvantaged students (56).

Counselling interventions likely operate at similar system levels as mentoring and family involvement, aiming to improve engagement through support. However, the accreditation programme also emphasises improving school culture, engaging both the mesosystem and exosystem through broader school policies and environments.

It is unclear whether the observed effects are linked to changes in school culture, as the study did not specify which accreditation components were implemented. The retrospective observational design limits the ability to establish causation, and there are concerns about potential bias from confounding factors. Nevertheless, the reductions in persistent absenteeism for Hispanic and economically disadvantaged students are notable, as these groups are known to be at higher risk of absenteeism (23, 81), making such interventions valuable.

This review found weak to moderate evidence suggesting that students' use of school-based healthcare (SBHC) may be associated with lower absenteeism for at-risk students. However, no clear causation could be established, and differences in healthcare access between the US and UK must be considered.

One study linked healthcare clinic outcomes with other strategies in a multi-faceted approach, while another retrospective observational study had high risks of confounding and attrition bias. Additionally, the second study was conducted over 30 years ago and may reflect outdated practices, such as recommending bedrest, which is a rare approach in modern medical practice. The remaining two studies were both retrospective, focusing on mental health services and prenatal care in school clinics (57, 64). These also faced confounding risks, with different rates of absenteeism and health status between groups at baseline.

In the bioecological model, SBHC impacts a student's microsystem through direct healthcare provision. Convenience and availability of care were considered key factors in included studies, alongside increased social support and positive role models, which reflect changes at the mesosystem level.

Existing literature indicates that the use of SBHCs, rather than simply their presence, can improve academic outcomes (82), and may also enhance school connectedness (83). However, the complexity of the mechanisms involved, including potential exosystem effects through health-positive school policies, requires further investigation.

One study suggested that delivering Social Emotional Learning (SEL) through a cognitive behavioural social skills curriculum may increase absenteeism. However, this finding was not consistently replicated across all time points, and the study's limitations (single-site, high risk of bias, confounding, and attrition) weaken the evidence (60). In this study, the curriculum was delivered to a class within a school with poor discipline and attendance. Improvements in self-reported behaviour were noted, which can be related to the students' mesosystem through peer relationships, influences in the school environment, and impacts on a students' sense of safety and belonging. The increased absences in the intervention group was unexpected, and is not replicated in other research. A previous meta-analysis evaluated the effect of universal school-based SEL interventions to find positive effects on similar social and emotional outcomes, alongside positive academic effects including improved attendance (84). They understood findings with a focus on environment, indicating the importance of universal implementation. This is in contrast to the intervention in this review, which was delivered to a single class. Delivery to a single class may overlook the benefits of continuity and synergy that can be achieved with wider implementation (85).

The authors identified existing norms and the schools' “expectation climate” as factors that undermined implementation. Despite the study limitations and the uncertainty about the effect on attendance, it is apparent that mesosystem factors, school culture and exosystem factors impacted both the implementation of the intervention and its effects.

A police partnership intervention led to a positive change in students' willingness to attend school, influencing all levels of the bioecological system. The intervention not only affected the microsystem (direct student action) and mesosystem (family and school involvement) but also extended to the exosystem through police presence, signalling legal support for school attendance. Additionally, the use of co-production and a procedurally just approach reflects a macrosystem change, aligning societal values with the need for legal compliance in education. Co-production fosters collaboration and more sustainable outcomes, and has been shown to be effective in tackling attendance issues (86). A multi-agency approach, including councils, healthcare providers, social workers, and community organisations, is key to overcoming barriers and improving support for at-risk students (87).

The final category of intervention studied was an incentive scheme, which resulted in an improvement in attendance from 82.4% to 92% (70). Students who did not receive family involvement participated in daily check-ins, celebrations, and rewards, applying behaviourist principles at the microsystem level through positive reinforcement (88). In this context, students with persistent absenteeism receive rewards for attending, which can create a motivating and encouraging environment. This approach contrasts with previous punitive measures they may have experienced due to absences, which can worsen attendance (89).

Additionally, group celebrations, viewed through social learning theory (90), encourage students to emulate positive behaviour observed in peers. While incentive schemes have had mixed results (30), they are valued for fostering a positive culture (91) and play a key role in absence prevention in multi-tiered strategies (34). However, the short study period and single-site design require caution in generalising outcomes and assessing long-term effectiveness.

### 4.2 Synthesis

**Figure 4:**
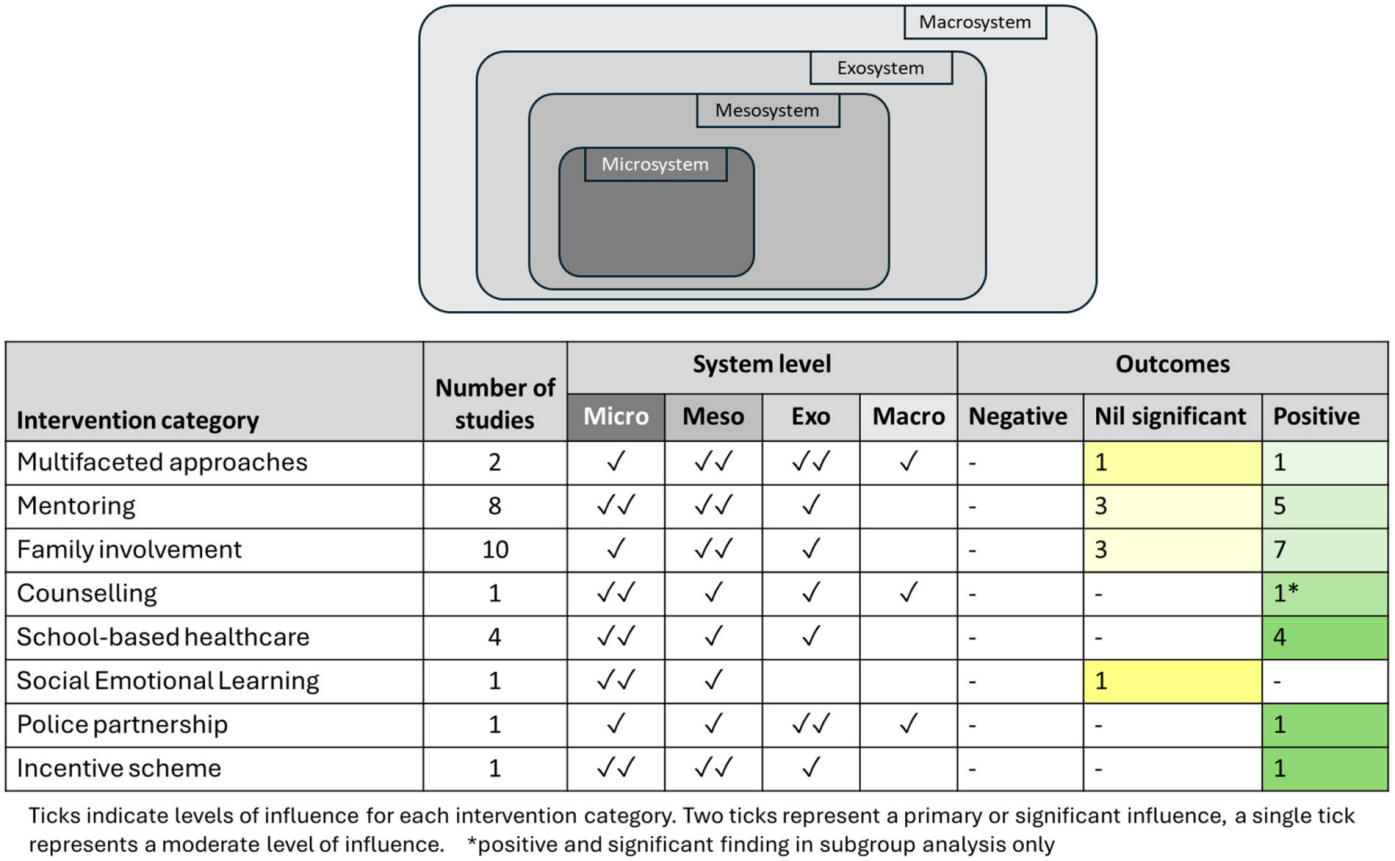
Included interventions mapped to KiTES bioecological framework.

The interventions across included studies were widely varied, and understanding links to outcomes is complex. Mapping interventions in the context of the KiTES bioecological framework (38) provides a basis to explore differences in direction and size of effects further. The included studies provide examples of interventions with influence across the bioecological system. Figure 4 provides an overview of results with application to the framework.

This summary highlights the lack of definitive conclusions regarding the application of these findings to a bioecological framework. There is no clear evidence that interventions must focus on one level of the system to be effective, nor a consistent correlation between engagement at multiple levels and effectiveness. While some multi-level interventions showed positive outcomes, others did not. Similarly, both comprehensive and targeted interventions have had mixed results, with some showing significant improvements in attendance and others having little or no effect. The effectiveness of strategies seems to depend on various factors, some within the scope of the interventions and others beyond it. What works in one context may not be effective in another.

### 4.3 Strengths

This review applied a systematic methodology, enhancing the reliability and validity of findings. The use of transparent reporting and adherence to established frameworks provides clarity in decision-making and allows for reproducibility. A narrow focus on secondary school students, particularly those at risk of persistent absenteeism, ensured that the findings were relevant and specific. This targeted approach allowed for more precise insights applicable to real-world educational settings.

The search strategy spanned both health and education databases, with expert input to ensure coverage of relevant literature. This breadth allowed identification of studies across multiple intervention types and school settings. Incorporating a bioecological framework for analysis provided a structured lens, helping to interpret the findings more coherently. Additionally, the narrative approach allowed for capturing nuances in the interventions that quantitative methods might have missed, offering policymakers valuable context.

The inclusion of various study designs, from randomised controlled trials (RCTs) to observational studies, helped strengthen the overall evaluation. Combining study type provided insight into both effectiveness and real-world implementation. While RCTs offered stronger internal validity, their findings were mixed: several mentoring trials showed only small improvements in attendance, and in some cases no statistically significant effect. In contrast, quasi-experimental and observational studies more frequently reported larger reductions in absence, particularly where interventions were embedded in broader school environments (e.g., school-based health centres or counselling programmes). However, these designs are more vulnerable to bias, making it difficult to determine whether improvements were due to the intervention or to other contextual factors. Bringing these approaches together allowed the review to balance certainty in effect estimates with understanding of how interventions operate in diverse educational settings, and highlighted that outcomes may depend heavily on local implementation, student needs, and school context.

### 4.4 Limitations

One of the main limitations of this review is the small evidence base, with most studies originating from the US and one from Australia. This limits generalisability to the UK context, especially considering cultural and policy differences. Absenteeism is recognised as a concern across Europe and other comparable education systems, but evidence remains limited internationally, as well as specifically in the UK. While a mix of study designs was used, the total number of studies (16) is insufficient to provide comprehensive insights into all intervention strategies within the bioecological framework.

There are also methodological limitations relating to the review process itself. The search strategy focused on school-based terms combined with “absen*” and related concepts. While this approach captured a wide range of studies addressing absenteeism, it may have missed interventions described solely using terms such as “persistent absence” without explicit reference to “school”. Additionally, terms such as “truancy” were not separately included, which may have limited retrieval of studies using older or justice-focused terminology. This is only partly mitigated with citation and reference searching.

Inclusion criteria required a measure of attendance for inclusion in this review; although there was no limitation on data type, a quantitative approach was usually adopted. Limited qualitative data may have overlooked deeper insights into the mechanisms behind interventions. Some studies failed to report key details on intervention integrity, such as frequency and duration, making comparisons difficult. The lack of detail in reporting limited the understanding of inconsistent findings, for example as seen with the implementation of Check & Connect (C&C) mentoring.

The heterogeneity of interventions presented a challenge in synthesis. Many programmes operated across more than one level of the bioecological model and contained multiple components spanning different intervention categories. As a result, some interventions were represented in more than one category. This reflects the inherently multi-component nature of attendance interventions rather than duplication of findings. However, it does mean the boundaries between categories should be interpreted as indicative rather than discrete. In some cases, separating components for categorisation risked oversimplifying how interventions functioned in practice, while retaining multi-level programmes within single categories could obscure important mechanisms of action. Although the synthesis aimed to balance capturing nuance with drawing out broader patterns, categories with only one or two interventions limit the strength of conclusions. A larger evidence base would allow more confident comparison within and across categories.

It is also important to note that the 10% threshold commonly used to define “persistent absenteeism” represents an administrative boundary rather than a distinct behavioural category. Interventions act both preventively, targeting pupils at risk of reaching this threshold, and responsively, supporting those already classified as persistently absent. Consequently, the included studies span a continuum of need, and effectiveness is likely to vary within that context.

Another concern is the risk of bias, which was moderate to high in several studies. This was mainly due to non-representative samples and significant dropout rates, common in populations at risk of absenteeism. Inconsistent attendance data and selective reporting further compounded the challenges in establishing the effectiveness of interventions. Additionally, conducting interventions in real-world settings introduced challenges such as recruitment and variable implementation, which further limits the generalisability of findings. Many studies also lacked detailed demographic reporting, such as ethnicity and socioeconomic status, which are known risk factors for absenteeism and can introduce bias into outcomes when unmatched.

As outlined in the introduction, similar quality findings have been reported in other reviews of school-based interventions using a range of quality appraisal tools. Reviews applying the EPHPP in comparable contexts have also found few studies rated as strong (92, 93). This suggests that the lack of high-quality studies reflects broader methodological constraints in evaluating real-world school-based interventions, particularly those involving at-risk or hard-to-engage populations, rather than an artefact of specific appraisal tools.

The process of study screening and appraisal also introduced potential bias. While quality appraisal was carried out systematically, only a small proportion of studies (3 out of 16, 18.8%) were independently assessed by a second reviewer. This falls short of gold-standard systematic review practice and may have influenced judgements of study quality.

Finally, where descriptions of interventions and their implementation was not reported in detail there was a need for interpretation. Conclusions are inherently shaped by judgements, which were in turn influenced by biases from author positionality.

### 4.5 Clinical & policy implications

This review offers valuable insights for policymakers in planning interventions to address persistent absenteeism in secondary school students. Several interventions showed positive impacts on attendance, with four studies improving attendance by over 5%, equivalent to more than 8 extra days in school over an academic year (54, 57, 62, 63). Additionally, two other studies showed improvements of over 2%, approximately 4 extra days (61, 68). These findings suggest that targeted interventions can make a difference, although the practical significance requires careful interpretation.

The meaning of any attendance change is highly context dependent. For some students, a few additional days in school may have limited academic impact, particularly where disengagement is deep-rooted. For others, such as those returning from emotionally based school avoidance or prolonged absence; small, incremental gains can reflect important steps toward re-engagement, trust-building, and reduced anxiety. Attendance outcomes should therefore be considered alongside baseline attendance, reasons for absence, level of need, and the nature of support provided.

Students at risk of persistent absenteeism often face challenges that also impact their ability to participate in interventions, which are in turn reflected in enrolment and attrition issues in research. Effective implementation planning should address these challenges to ensure interventions are accessible and have maximal impact. It's crucial to consider specific subgroups of students, including those with economic disadvantage, minority ethnic groups, and those with special educational needs. Interventions need to be tailored to address the unique barriers faced by these students.

Differential findings between intent-to-treat and treatment-on-treated groups emphasise the importance of implementation fidelity. Ensuring high-quality, consistent implementation is key to achieving programme success. Policy planning should include strategies for ensuring realistic yet ambitious implementation levels, including co-production, clear guidelines, robust training, and ongoing support.

### 4.6 Future research directions

This review highlighted the limited evidence on effective strategies for addressing persistent absenteeism in secondary schools. The scarcity of eligible studies and the lack of UK-based research underscore the need for more robust studies in this area. Persistent absenteeism continues to be a pressing educational and public health issue, presenting an opportunity to further investigate interventions with well-designed, high-quality research. Cost-effectiveness research will also be vital to guide resource allocation and ensure interventions benefit vulnerable populations.

There was limited evidence to understand the relationship between school culture, interventions that impact it, and their effect on student attendance. This reflects that many interventions may focus on broader objectives such as school culture, rather than directly addressing persistent absenteeism. In the full-text review, almost 30% of the studies (41 out of 138) were excluded due to not focusing specifically on persistent absenteeism or at-risk students. Future reviews could benefit from broader inclusion criteria to capture the full scope of cultural interventions, especially given the UK's focus on promoting a safe, supportive learning environment in schools. More primary research is needed to explore how interventions that aim to improve school culture specifically impact students with persistent absenteeism.

Future research should incorporate longer follow-up periods and consider outcomes beyond attendance alone, including attainment, wellbeing, and post-16 transitions. Understanding whether any change is maintained, and under what conditions, is essential for informing long-term policy and resource planning.

Evaluations should account for baseline attendance, reasons for absence, level of need, and demographic and contextual factors, including culturally specific barriers, accessibility, and economic influences. Demographic subgroups should be recorded, and qualitative research could provide valuable insights into designing more tailored interventions. This would include understanding culturally specific barriers, improving accessibility, and addressing economic factors that influence attendance.

The involvement of students experiencing persistent absenteeism in the development and refinement of interventions was also largely absent from the studies reviewed. Given the complex and individual nature of school avoidance, co-design approaches involving young people, families, and school staff could help ensure that interventions are acceptable, relevant, and responsive to lived experience. Future research and programme design including participatory or co-production methods can support more tailored and impactful interventions.

The application of the bioecological framework in this synthesis provided a useful basis for exploration of intervention efficacy and underlying mechanisms, although limited. Adopting this approach with a larger evidence base in future research can establish a deeper understanding of how interventions interact with different levels of the bioecological model to influence persistent absenteeism.

Finally, future research should focus on the quality, depth, and role of intervention components, rather than trying to separate them artificially, as students' needs are interconnected and require multifaceted responses. In particular, examining how specific components function would strengthen understanding of how interventions work in practice, for example, the way family involvement ranges from one-way communication to genuine partnership. Realist evaluation approaches could support this by exploring what works, for whom, and in what contexts, helping to clarify mechanisms and inform more tailored, impactful interventions.

## 5 Conclusion

This review provides limited evidence for the effectiveness of interventions aimed at reducing persistent absenteeism in secondary school students. While mentoring schemes, family involvement, school counselling, and other approaches show potential, their success is not guaranteed, and the findings are compromised by methodological issues and inadequate reporting. The evidence is further limited by a small number of studies, all from the US or Australia, which may not be directly applicable to a UK context.

Given the low quality of evidence and significant risks of bias, further research is necessary to assess the effectiveness of these interventions, particularly in the UK. Future studies should focus on robust designs, clearer reporting, and a better understanding of how different intervention levels (individual, family, school, community, societal) compare in terms of impact. There is also a need to explore the role of school culture more deeply.

In conclusion, while some interventions show promise for students with persistent absenteeism, including those experiencing emotionally based school avoidance, further rigorous, context-specific research is needed to develop strategies that can be confidently recommended.

## Data Availability

The original contributions presented in the study are included in the article/Supplementary Material, further inquiries can be directed to the corresponding author.
