# Supplementary figures and images for: What school-based interventions work to improve attendance in secondary school students with persistent absence? A systematic review
Source: Front Child Adolesc Psychiatry. 2026 Jan 12;4:1603680. doi: 10.3389/frcha.2025.1603680 (PMC12832960; doi:10.3389/frcha.2025.1603680)

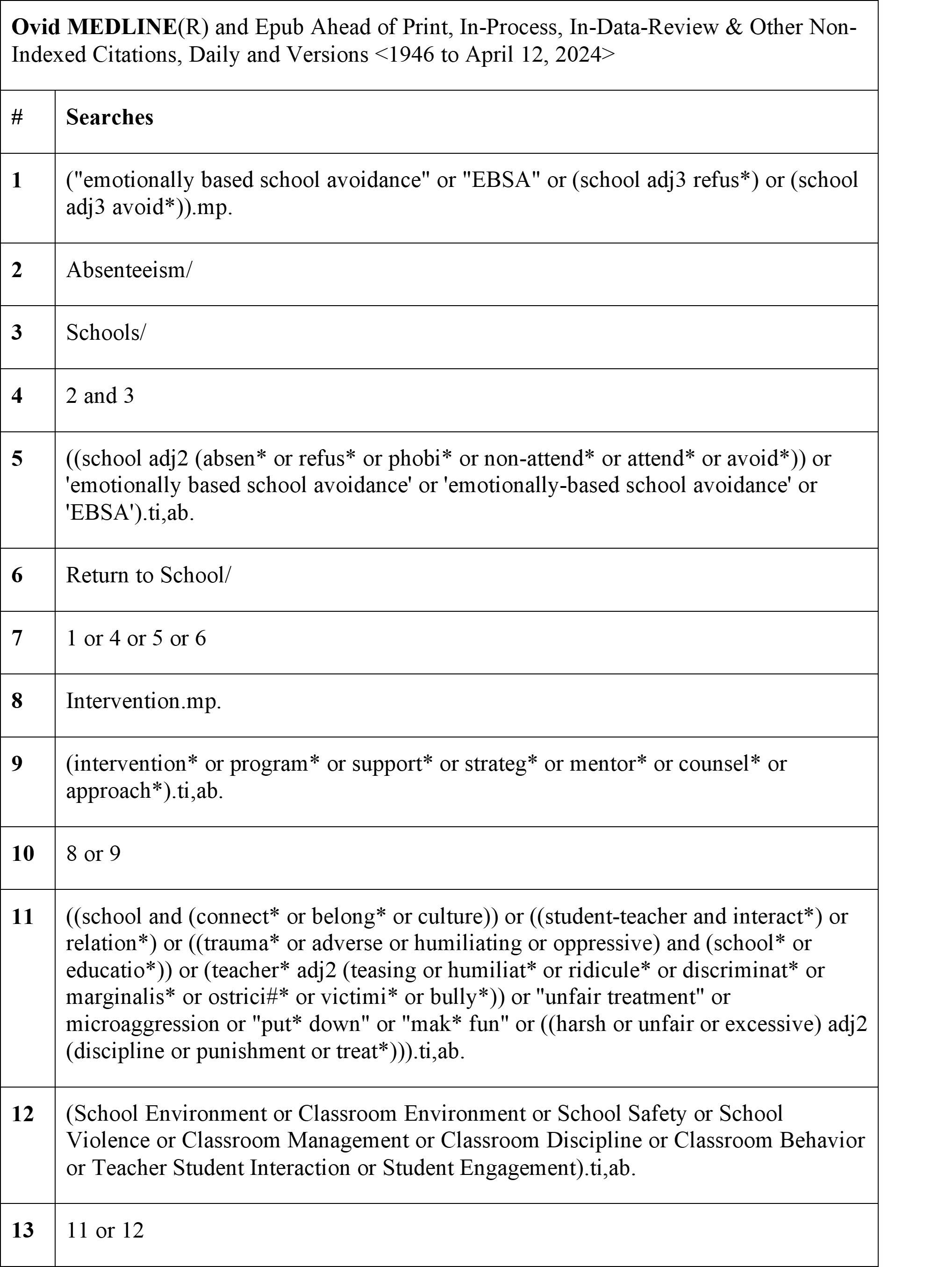

Supplement: Supplementary file 1 [file Supplementaryfile1.png]
